# Contributions of capsule, lipoproteins and duration of colonisation towards the protective immunity of prior *Streptococcus pneumoniae* nasopharyngeal colonisation

**DOI:** 10.1016/j.vaccine.2012.04.080

**Published:** 2012-06-22

**Authors:** Jonathan M. Cohen, Suneeta Chimalapati, Corné de Vogel, Alex van Belkum, Helen E. Baxendale, Jeremy S. Brown

**Affiliations:** a Centre for Respiratory Research, Department of Medicine, UCL, London, United Kingdom; b Infectious Diseases & Microbiology Unit, UCL Institute of Child Health, London, United Kingdom; c Department of Medical Microbiology and Infectious Diseases, Erasmus Medical Centre, Rotterdam, The Netherlands

**Keywords:** *Streptococcus pneumoniae*, Colonisation, Antibody, Capsule, Auxotroph, Lipoprotein

## Abstract

Live attenuated vaccines have been proposed as a strategy to induce protective immunity against infectious diseases. Recent data have demonstrated that nasopharyngeal colonisation with *Streptococcus pneumoniae* induces protective immunity against subsequent invasive infection, suggesting nasal vaccination with live attenuated bacteria could be a preventative strategy. However the bacterial factors affecting the strength of this adaptive immune response remain unclear. In a direct comparison with the parent wild-type strain, we found that colonisation with bacteria lacking either capsule or surface lipoproteins led to significantly diminished protection. Immunity after colonisation was not dependent on serum IgG to capsular antigens. Colonisation density and duration was reduced for all the non-protective strains, suggesting that protective immunity maybe related to the extent of nasopharyngeal bacterial exposure. To investigate this hypothesis, we utilised an auxotrophic bacterial Δ*pab* strain where duration of colonisation could be controlled by supply and removal of para-amino-benzoic acid (PABA) to mouse drinking water. Supporting colonisation with the Δ*pab* strain for 5 days with PABA led to a faster serum antibody response compared to colonisation for less than 48 h. This enhanced immunogenicity was associated with a trend towards protection. The data presented here aid our understanding of why only certain live attenuated strains are able to function as effective vaccines, and may be valuable in informing the constituents of future live attenuated vaccines.

## Highlights

► Live attenuated nasopharyngeal *S. pneumoniae* exposure protects against invasive disease. ► Presence of capsule and lipoproteins during colonisation enhances immunogenicity and protection. ► Colonisation does not induce serum anti-capsular IgG. ► Duration/density of colonisation is important in determining immunogenicity of bacteria. ► Inclusion of capsule may enhance protective immunity of otherwise attenuated strains.

## 1 Introduction

*Streptococcus pneumoniae* is a global pathogen responsible for the deaths of over one million individuals annually, mostly due to pneumonia [1]. Initial exposure to the bacteria is in the nasopharynx, where they establish colonisation. Usually, episodes of nasopharyngeal colonisation are essentially asymptomatic, and do not lead to disease [2]. In certain cases however, when the range of innate and adaptive immune mechanisms is insufficient to prevent disease, aspiration of bacteria can lead to pneumonia. This is most common at the extremes of life and amongst immunocompromised individuals. Vaccines have been directed to this specific need.

At present, licensed vaccines elicit protection through induction of opsonophagocytic antibodies against capsular polysaccharide antigens [3]. Once conjugated to carrier proteins, a process necessary to induce protection in infants, these vaccines can lead to reduction in carriage as well as disease. These conjugate vaccines are very effective at reducing disease caused by the *S. pneumoniae* serotypes included in the vaccine directly in the vaccinees and indirectly in the wider community. However, serotypes not included in the vaccine can replace the eliminated strains within the nasopharynx, leading to replacement disease [4]. Despite recent increases in the number of serotypes included in vaccine formulations, it is likely that alternative strategies will be required in the long-term to protect against *S. pneumoniae* [3].

Live vaccines can lead to both humoral and cellular immune responses. Inclusion of a large number of antigens and natural bacterial adjuvants can lead to strong immunity in the absence of an exogenous adjuvant. Nasopharyngeal colonisation with live bacterial strains represents one such route of mucosal immunisation.

Using murine models, we [5] and others [6,7] have studied the mechanisms by which prior colonisation can protect against subsequent lethal invasive pneumonia. Antibody responses induced through colonisation with a live wild-type (WT) strain are both necessary and sufficient to protect against invasive disease [5]. Such protection does not necessarily require antibodies to capsular polysaccharide, since experimental colonisation with unencapsulated strains is also protective [6]. Unencapsulated mutants are an attractive option for live attenuated vaccines due to their lack of virulence [6,8], but no direct comparison of the immunogenicity and protective efficacy of colonisation with isogenic strains with and without capsule has been reported.

Bacterial lipoproteins are an important class of pathogen-associated molecular pattern (PAMP), capable of adjuvanting immune responses [9] by acting as ligands for TLR2 [10], and are common targets for adaptive immune responses [11,12]. Deletion of *lgt*, which encodes the protein diacylglyceryl transferase required to anchor lipoproteins to the cell membrane, results in an *S. pneumoniae* strain with greatly reduced quantities of lipoproteins on the bacterial surface (Chimalapati, under review) [13]. This Δ*lgt* strain is still able to colonise the mouse nasopharynx, albeit with both reduced density and shorter duration than its parent WT strain. Its ability to induce protective immunity is not known.

The gene *pabB* encodes para-amino benzoic acid (PABA) synthase, required for the folate biosynthetic pathway. Deletion of this gene leads to an auxotrophic mutant where growth is dependent upon exogenous supply of PABA [11]. It is unlikely to affect capsule expression since phagocytosis of the Δ*pab* strain *in vitro* is similar to that of its parent strain [11]. The Δ*pab* mutation does not significantly effect lipoprotein expression, since such strains can robustly induce anti-lipoprotein antibodies when inoculated *via* the intraperitoneal route [11]. This mutation results in an inability to replicate *in vivo*, and was previously reported to lead to rapid clearance of TIGR4Δ*pab* from the nasopharynx within 2 days. This mutant was also avirulent unless the animal's drinking water was supplemented with PABA [11]. Again, its ability to induce protection through colonisation is not known.

In this study, we address the specific contribution of the presence of capsule and surface lipoproteins on colonisation-induced immunogenicity and protection against subsequent lethal pneumonia. We find that absence of either capsule or lipoproteins leads to failure to protect, reflecting reduced immunogenicity. Using controlled colonisation with an auxotrophic mutant, we find that duration and density of colonisation directly impacts on the speed of the immune response, with potential impact on subsequent protection.

## 2 Methods

### 2.1 Ethics statement

Experiments were approved by the UCL Biological Services Ethical Committee and the UK Home Office (Project Licence PPL70/6510). Experiments were performed according to UK national guidelines for animal use and care, under UK Home Office licence and in accordance with EU Directive 2010/63/EU.

### 2.2 Bacterial strains and culture conditions

Wild-type (WT) *S. pneumoniae* strain D39 (serotype 2) and its unencapsulated derivative containing a deletion of *cpsD* (D39-DΔ) [14] were a kind gift from James Paton, University of Adelaide. Deletional mutant strain D39Δ*pab* lacking PAB synthetase or *lgt* were generated by overlap extension PCR as described [11] (Chimalapati, under review). Bacteria were cultured on Columbia agar with 5% horse blood or in Todd–Hewitt broth with 0.5% yeast extract in 5% CO_2_. Inocula for challenge experiments were prepared from mid-log phase cultures and stored at −70 °C as single use aliquots.

### 2.3 Colonisation and infection models

CD1 outbred mice were obtained from Charles River UK Ltd. Mice were colonised by instillation of 10^7^ cfu *S. pneumonia* in 10 μl PBS into the nares under light halothane anaesthesia as previously [5,15]. In certain experiments, mice received a second colonising dose 2 weeks after the first dose. Control mice received 10 μl PBS alone. To obtain nasal washes the exposed trachea was flushed caudally with 200 μl PBS and the fluid exiting the nares collected. For the pneumonia challenge, 10^7^ cfu *S. pneumonia* D39 in 50 μl PBS was instilled into the nares under deep general halothane anaesthesia 28 days after the final colonising dose [5,15,16]. Animals were culled by exsanguination from the femoral artery under pentobarbital anaesthesia. Broncheo-alveolar lavage fluid (BALF) was collected by cannulating the exposed trachea and washing the airways three times serially with 1 ml sterile PBS. Lungs were collected aseptically into ice-cold PBS, minced and homogenised with sterile PBS as previously [5,17]. For survival experiments, animals were monitored and culled when exhibiting previously defined features of terminal disease [16].

### 2.4 Whole cell ELISA

Antibodies specific to antigens in different *S. pneumoniae* strains were measured by whole cell ELISA using established methods as previously described [8]. Briefly, *S. pneumoniae* were grown to late log-phase, washed and resuspended in PBS to OD_580_ 1.0. 96-well plates were coated with this bacterial suspension, refrigerated overnight, then blocked with PBS 1% BSA prior to use. Sera were diluted in PBS 1% BSA before addition and binding to bacterial antigens detected with anti-mouse secondary antibodies conjugated to alkaline phosphatase (Sigma).

### 2.5 Capsule ELISA

To measure capsule-specific antibodies, plates were coated with type 2 purified capsular polysaccharide (CPS) at 10 μg/ml (LGC Promochem). To increase assay specificity, sera were pre-incubated with cell wall polysaccharide (Statens Serum Institut) and type 22F capsular polysaccharide (LGC Promochem) as previously [11]. Development of ELISAs proceeded as for whole cell ELISAs.

### 2.6 Competitive inhibition ELISA

To determine the relative contribution of CPS binding towards the total binding observed in whole cell ELISA, sera were pre-incubated in PBS/1% BSA with increasing concentrations of soluble type 2 CPS up to 100 μg/ml for 30 min at RT, prior to assay in whole cell ELISA as above.

### 2.7 Luminex anti-pneumococcal protein antibody assay

Antibody responses to multiple protein antigens were measured using a multiplex flow cytometry Luminex assay based on *S. pneumoniae* proteins conjugated to xMAP beads, as previously [11]. Recombinant TIGR4-, D39-, or serotype 23 strain-derived proteins were conjugated to xMAP beads (Luminex) [18]. Combined beads (3000 per antigen) were incubated with 10% or 1% serum in PBS–1% bovine serum albumin and then with goat anti-mouse IgG-phycoerythrin (Jackson ImmunoResearch). IgG binding was subsequently assessed using a Bioplex instrument (Bio-Rad Labs) and Bio-Plex Manager software. Data are presented as log_10_ MFIs of IgG binding to each bead type, after subtraction of the results for blank beads. There was no binding to proteins using serum from control mice.

### 2.8 Statistics

Bacterial loads were compared at specific time-points by Mann–Whitney *U*-test. Antibody levels were compared between groups of mice by two-tailed Student's *t*-test. Survival of challenged mice was compared by the log rank test. *P* values <0.05 were considered significant.

## 3 Results

### 3.1 Protection against lethal pneumonia challenge

**Fig. 1:**
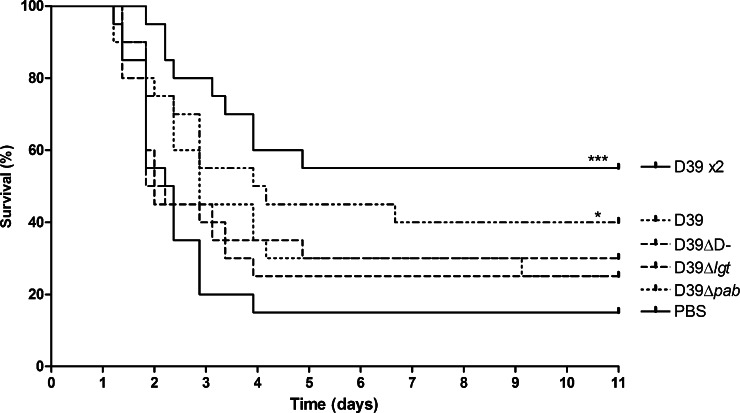
Protective effect of prior colonisation on subsequent *S. pneumoniae* pneumonia. Kaplan–Meier survival curves of mice challenged with 10^7^ cfu of D39 i.n., 28 days after colonisation with either one or two (×2) doses of 10^7^ cfu of D39, D39-DΔ, D39Δ*lgt*, D39Δ*pab* or PBS alone (*n* = 18–20). *P < 0.05, ***P < 0.001.

To assess the protection induced through colonisation with wild-type D39, unencapsulated D39-DΔ, lipoprotein-deficient D39Δ*lgt* or auxotrophic D39Δ*pab*, groups of 20 outbred CD1 mice were colonised with 10^7^ cfu of these strains. A further group received 2 colonising doses of 10^7^ cfu D39, 2 weeks apart. A control group received PBS in place of bacterial colonisation. All mice were challenged nasally at the same time, 28 days following final colonisation, with 10^7^ cfu WT D39 (Fig. 1). In addition, serum was also collected from 10 mice per group the day prior to challenge. In this invasive pneumonia model, challenge led to septicaemia with death of the majority of control mice (15% survival), with a median survival of 2.29 days. Mice previously colonised with D39 WT were protected against challenge with a survival of 40% (group median survival time 4.04 days, *P* = 0.003). Amongst mice that received 2 colonising doses of D39, survival was improved at 55% (*P* = 0.001). However, mice colonised with the mutant strains were not significantly protected, with survival rates of 30% (median survival 2.02 days) in mice colonised with D39-DΔ, 25% (median survival 2.0 days) in mice colonised with D39Δ*lgt* and 25% (median survival 2.87 days) in mice colonised with D39Δ*pab*.

### 3.2 Immunogenicity following colonisation

**Fig. 2:**
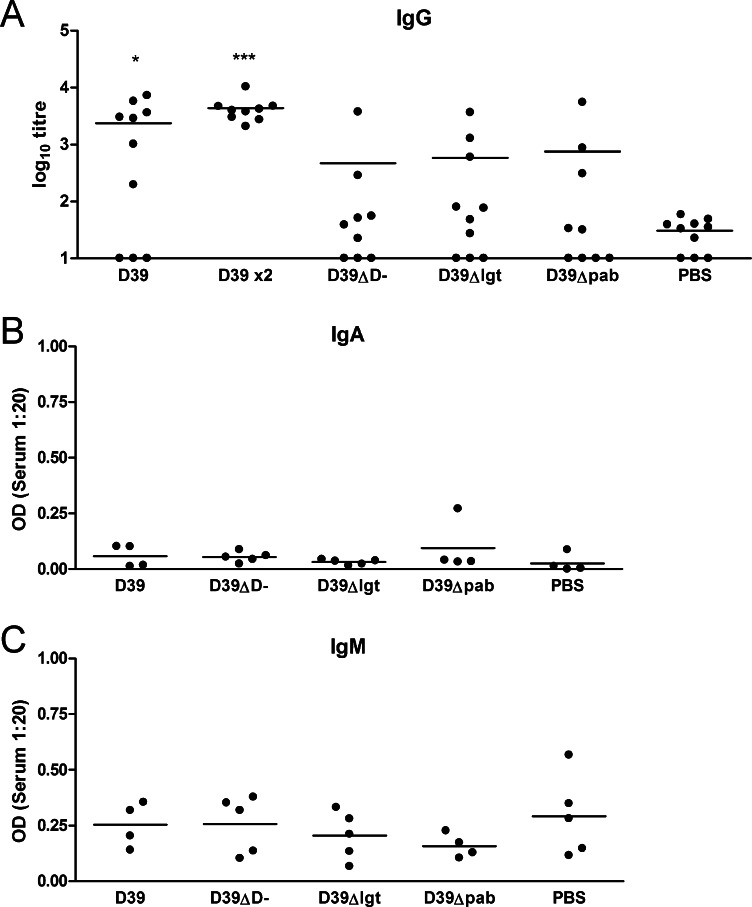
Serum antibody response to colonisation. Serum IgG (A), IgA (B) and IgM (C) responses to colonisation with either one or two (×2) doses of D39, D39-DΔ, D39Δ*lgt* or PBS alone, measured using whole cell ELISA. Dots indicate responses of individual mice, bars represent mean for the group. *P < 0.05, ***P < 0.001.

**Fig. 3:**
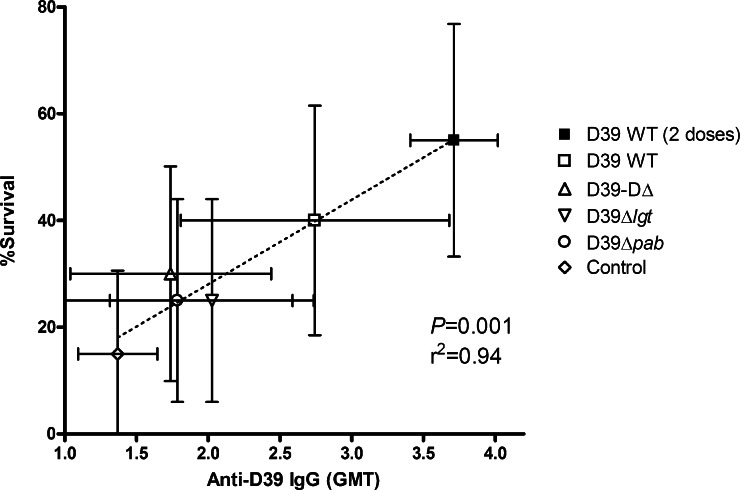
Correlation of immunogenicity with protection following nasopharyngeal colonisation. Coloured dots and bars represent immunogenicity as anti-D39WT IgG geomean titre (GMT) of the group (±95% CI) *versus* percentage survival (±95% CI) of the group against day 28 D39WT pneumonia challenge. Dotted line shows correlation between immunogenicity and protection by linear regression.

The lack of protection afforded with D39-DΔ, D39Δ*lgt* or D39Δ*pab* in this model suggested that colonisation with these strains was insufficiently immunogenic to protect against invasive pneumonia. To test this, antibody was measured in individual sera from colonised and control mice. Antibodies to total bacterial antigens were measured by whole cell ELISA (Fig. 2). 70% of mice colonised with D39 developed an IgG ELISA titre response to D39 greater than the level observed in control mice which had been sham colonised with PBS. This increased to 100% in mice receiving two doses. Only in mice colonised with the wild-type strain were IgG levels significantly higher than those observed in controls. In groups receiving unencapsulated D39-DΔ, lipoprotein-deficient D39Δ*lgt* or auxotrophic D39Δ*pab*, less than 50% of mice developed anti-D39 IgG titres greater than that seen in controls. There was no evidence for significant anti-D39 IgA or IgM responses by day 28 post-colonisation with any of the strains. The degree of protection against invasive pneumonia challenge afforded by the different strains correlated strongly with the levels of serum anti-D39 IgG (*r*^2^ = 0.94, *P* < 0.001) (Fig. 3). These responses are in accordance with the immunogenicity of D39 colonisation in inbred CBA/Ca mice [5], where protection is known to be mediated by serum IgG.

**Fig. 4:**
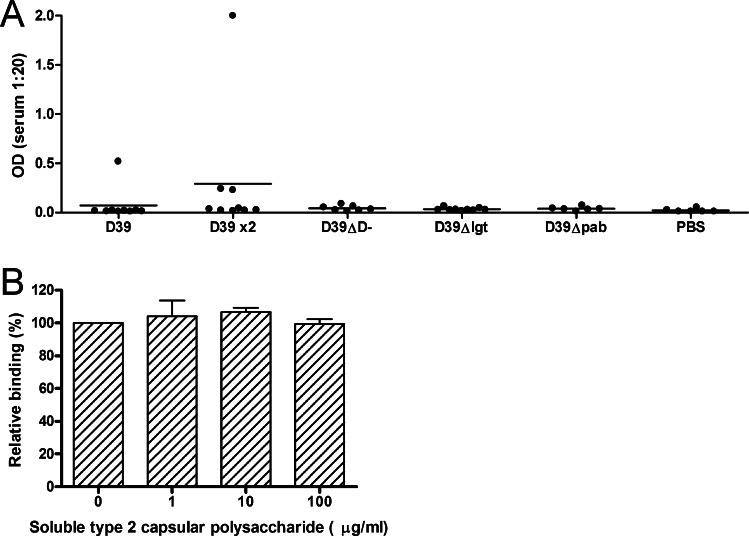
Serum anti-capsular IgG response to colonisation. (A) Serum anti-type 2 capsular polysaccharide IgG response to colonisation with either one or two (×2) doses of D39, D39-DΔ, D39Δ*lgt*, D39Δ*pab* or PBS alone, measured by ELISA. Dots indicate responses of individual mice, bars represent mean for the group. (B) Competitive inhibition of binding of serum IgG to total D39 antigens in whole cell ELISA using increasing concentrations of soluble type 2 capsular polysaccharide. Bars represent relative mean ± SD of binding of sera from individual mice (*n* = 4).

**Fig. 5:**
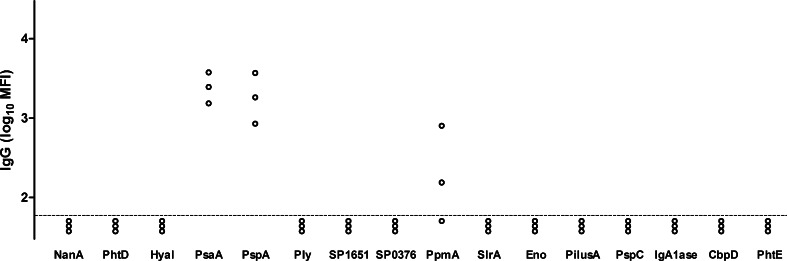
Serum anti-protein IgG responses to colonisation. IgG binding of sera of three individual WT D39 colonised mice to *S. pneumoniae* proteins measured by Luminex bead assay. Dotted line represents limit of detection.

Colonisation with an unencapsulated mutant of a type 6A strain of *S. pneumoniae* can induce protection against challenge with the encapsulated parent WT strain [6]. We were therefore surprised that D39-DΔ was poorly immunogenic in our model. We initially hypothesised that protection induced through colonisation with the wild-type strain was mediated through anti-capsular antibody. If so, lack of this critical antigen would explain the limited immunogenicity of the unencapsulated strain. Serum anti-type 2 capsular polysaccharide IgG was measured by ELISA (Fig. 4A). Whilst nearly all mice colonised with WT D39 developed an IgG response as measured in whole cell ELISA (Fig. 2A), only an occasional mouse developed a capsule-specific IgG response (Fig. 4A). Anti-CPS IgG made a negligible contribution to total IgG binding as assayed by whole cell ELISA since pre-incubation of sera with excess purified capsular polysaccharide antigen did not inhibit IgG binding in sera from mice colonised with WT D39 (Fig. 4B). To further confirm that colonisation with WT D39 induced antibody against non-capsular antigens, levels of IgG that bound to pneumolysin and 15 surface-accessible protein antigens was measured in the serum of 3 randomly selected WT D39 colonised mice (Fig. 5). Antibody to pneumococcal surface protein A (PspA) and the lipoprotein pneumococcal surface adhesin A (PsaA) were detected in 3 out of 3 mice, and IgG to the lipoprotein putative proteinase maturation protein (PpmA) in 2 of 3 mice. Thus, colonisation with the encapsulated WT strain induced antibody to bacterial proteins including lipoproteins, but not to capsular polysaccharide. Colonisation with either D39-DΔ or D39Δ*lgt* was less immunogenic, correlating with their lack of protection.

### 3.3 Density and duration of colonisation

**Fig. 6:**
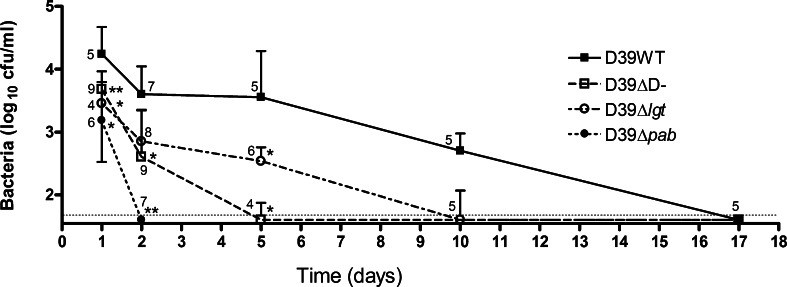
Timecourse of colonisation with *S. pneumoniae* D39 strains. Bacterial cfu recovered from nasal washes of CD1 mice on days 1, 2, 5, 10 and 17 following intranasal inoculation in 10 μl of PBS containing 10^7^ cfu D39 *S. pneumoniae* (closed squares) or D39-DΔ (open squares), D39Δ*pab* (closed circles) or D39Δ*lgt* (open circles). Median and interquartile range of groups of 4–9 mice at each timepoint are shown, along with the precise number in the group. The dotted line is the limit of detection.

Since neither D39-DΔ and D39Δ*pab* lacked the potentially protective antigens present in WT D39, we generated the alternative hypothesis that lack of protection reflected insufficient antigen exposure during the colonisation process. To explore this, we compared the density and duration of nasopharyngeal colonisation with these strains (Fig. 6). D39 colonisation persisted until at least day 10 following inoculation, but no bacteria were recovered by day 17. The ability of D39-DΔ to colonise was impaired. Compared to WT, there were approximately 1-log fewer unencapsulated D39-DΔ recovered at both day 1 and day 2 post-inoculation, with colonisation cleared in nearly all mice by day 5. As seen previously with TIGR4Δ*pab* [11], D39Δ*pab* bacteria were rapidly cleared within 48 h of attempted colonisation. We also found that D39Δ*lgt* has a shorter duration of colonisation (cleared by day 10) and lower colonisation density (approximately 1–1.5 log_10_ fewer) compared to WT D39 (data from Chimalapati et al., under review) (Fig. 6). Thus, the immunogenicity of the protective WT strain may reflect contributions by both capsule and surface lipoproteins to maintaining the degree of bacterial nasopharyngeal exposure required to induce protective immunity.

### 3.4 Effect of duration of colonisation on immune response

**Fig. 7:**
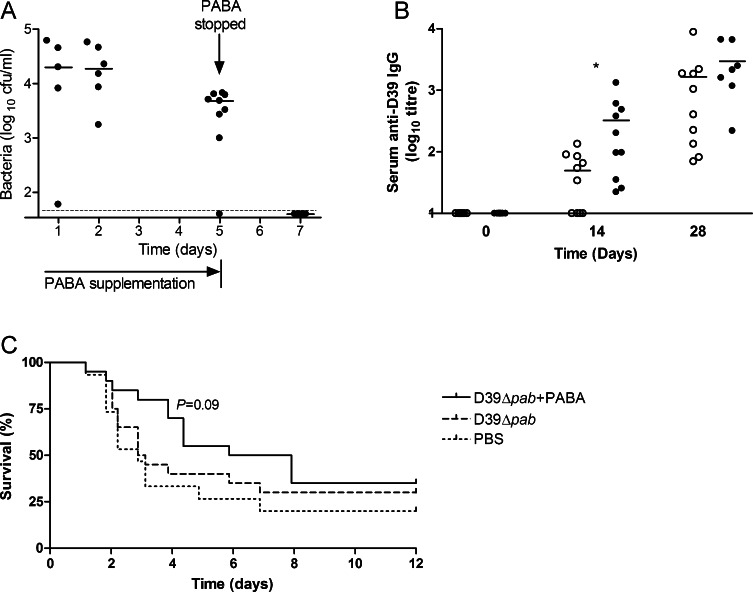
Effect of para-aminobenzoic acid (PABA) supplementation on immunogenicity and protection. (A) Bacterial cfu recovered from nasal washes on days 1, 2, 5 and 7 following intranasal inoculation with 10^7^ cfu D39Δ*pab* in the presence of PABA supplementation. PABA was withdrawn on day 5. Dots represent results for individual mice, bards represent group median. The dotted line is the limit of detection. (B) Serum anti-D39 IgG level measured by whole cell ELISA at 0, 14 and 28 days following colonisation with (black circles) or without (white circles) 5 days of PABA supplementation. Bars indicate group mean. *P < 0.05. (C) Survival following challenge with 10^7^ cfu D39 i.n. 28 days after colonisation with D39Δ*pab* with or without 5 days of PABA supplementation, or with PBS alone (*n* = 15).

To assess whether the duration of bacterial colonisation could be controlled using PABA supplementation of this mutant, we attempted to colonise mice with D39Δ*pab* in the presence of PABA supplementation. PABA supplementation was commenced the day prior to colonisation, and abruptly withdrawn after 5 days (Fig. 7A). In the presence of PABA, numbers of D39Δ*pab* were maintained in the nasopharynx at a level similar to that seen with WT D39. When withdrawn on day 5, bacterial numbers rapidly fell, and were no longer detectable after 48 h, by day 7 post-colonisation.

To investigate the impact of controlled colonisation on immunogenicity and protection, further groups of mice were colonised with D39Δ*pab* in the presence or absence of PABA for 5 days. Serum anti-D39 IgG level was assessed at 14 and 28 days, prior to pneumonia challenge with WT D39. By day 14 post-colonisation, mice receiving 5 days of PABA supplementation had approximately 10-fold greater median serum IgG against D39 than those not receiving PABA (Fig. 7B). By day 28, levels were not significantly different between the groups, indicating more rapid development of an antibody response when growth of the auxotrophic bacteria was supported at this level. In mice colonised with D39Δ*pab* alone, there was no evidence of protection (median survival time 3.00 days, overall survival 30%) compared to controls (median time 2.87 days, 20% survival) (Fig. 7C). In mice where colonisation was supported with PABA, there was a trend towards longer survival compared with controls (median survival time 6.90 days, overall survival 35%, *P* = 0.09). Thus, the enhanced immune kinetics suggested that the degree of nasopharyngeal bacterial exposure was directly impacting on subsequent immunogenicity, and could make a contribution towards protection.

## 4 Discussion

Live attenuated vaccines must possess both antigens and adjuvants which persist in sufficient quantity in an appropriate location for enough time to induce a protective response. We have investigated how multiple factors may contribute towards the immunogenicity of a colonising bacterial strain and determine whether the colonisation event is sufficient to induce protection. We have previously shown prior colonisation protects against invasive D39 pneumonia by preventing septicaemia with no protection at the mucosal level and is dependent on serum antibody [5]. Hence, systemic IgG rather than local immunological responses to colonisation are likely to be the important protective response for this model of *S. pneumoniae* infection, and this was supported by the close correlation between the serum IgG response and protective efficacy for the different strains studied here.

Compared to its WT parent strain, D39Δ*pab* was poorly immunogenic following colonisation. Supplementation with PABA for 5 days restored the ability of D39Δ*pab* to colonise, and enhanced the speed of anti-D39 IgG seroconversion. The majority of mice with PABA supplementation had high titres of anti-D39 IgG, whereas in mice without PABA titres were much more variable. This was associated with a strong trend towards protection. These data support the hypothesis that for a given strain of *S. pneumoniae*, the duration of colonisation is important in generating protective immunity. Whether the ‘area under the curve’ (reflecting total antigen present over time i.e. abundance as well as duration of colonisation) is more important than duration alone is not clear. Unencapsulated and *pspA*/*ply* mutants have been reported which also have shorter duration of colonisation at lower densities than the parent WT strain [6]. These were however still able to induce protective immune responses in C57BL/6 mice [6]. This may reflect a greater propensity to induce stronger protection in this inbred strain, which may explain the greater protection seen following WT D39 colonisation of CBA/Ca mice [5] than the CD1 mice reported here. It may be more challenging to achieve protection in outbred mice due to multiple genetic differences between individual mice including the MHC. Protection has been shown for a pneumolysin-deficient D39 strain in outbred MF1 mice [7], but colonisation with this strain persisted for 7–14 days and was not dissimilar to the duration of WT D39 in CD1 mice reported here.

Colonisation with the WT D39 strain induced high titres of anti-bacterial serum IgG, yet no detectable anti-capsular IgG. This was also found following D39 colonisation of CBA/Ca mice [5] and MF1 mice [7]. We have also found that colonisation of CD1 mice with the TIGR4 strain did not induce anti-capsular serum IgG (unpublished data). Together, these data suggest that, in mice, a single nasopharyngeal colonisation event is not sufficient to induce a serum anti-CPS IgG response, at least for serotype 2 and 4 capsules. Colonisation has a variable effect on induction of serum anti-CPS IgG responses in humans. In a longitudinal family study, serotypes 9V, 14, 18C, 19F and 23F induced anti-CPS responses, but serotype 6B did not [19]. Following carriage in a childhood study, responses were detected to serotypes 11A and 14, but not to serotypes 6B, 19F and 23F [20]. Furthermore, experimental human colonisation did not induce an anti-capsular serum IgG response [21]. Immunogenicity of capsule following colonisation events is likely to reflect a complex interaction of bacterial strain, CPS type, host genetics, as well as the current and previous constituents of the nasopharyngeal microbiome. Ongoing longitudinal studies correlating detailed carriage history with serological data may elucidate this further.

The absence of anti-capsular serum IgG did not prevent colonisation with WT D39 from inducing protection against lethal challenge, albeit at a weaker level in these CD1 mice compared to results with in-bred strains [5]. Immunity to non-capsular antigens induced through colonisation is known to be sufficient to protect [6]. Our data imply that whilst capsular antigens are not dominant during colonisation, the presence of capsule does not impede the development of anti-protein mediated protective immunity. On the contrary, the increased level and duration of colonisation with encapsulated compared to unencapsulated bacteria resulted in an increased antibody response to protein antigens and improved protection to subsequent challenge. Thus, for the strains tested here, the potentially stronger immunogenicity due to greater exposure of surface proteins in the unencapsulated strain is offset by shorter less-dense colonisation, rendering the strain non-protective. In this regard, it would be interesting to directly compare the immunogenicity and protective efficacy of colonisation with unencapsulated strains that are known to protect [6] with those of their WT parent strains. It is possible that WT strains in general would emerge as more immunogenic than unencapsulated isogenic mutants.

The reduced immunogenicity of the Δ*lgt* mutant is likely to reflect a combination of factors. Most important of these may be the reduced colonisation density and duration. In addition, colonisation with WT D39 induced serum IgG to only 3 of 16 proteins antigens tested and two of these three were lipoproteins. Thus if the antibodies binding these antigens makes a critical contribution to protection of the WT strain, the absence of the antigens in D39Δ*lgt* would significantly impair its ability to protect. TLR2 signalling is important in the induction of Th17-cell responses through *S. pneumoniae* colonisation. Thus, mice lacking TLR2 have delayed clearance of *S. pneumoniae* [22,23]. Reduced TLR2 signalling from D39Δ*lgt* may therefore impair the induction of the Th17 response and could reduce the immunogenicity of the Δ*lgt* strain. However, data from TLR2 deficient mice suggest that this pathway may be redundant in the induction of robust serum IgG responses to colonisation [24], perhaps due to other compensating pathogen recognition pathways. Similarly, TLR4 [25] and inflammasome [26,27] activation by pneumolysin may also be redundant in this regard, since pneumolysin-deficiency bacteria are also capable of inducing protection [7], perhaps due to intact TLR2 signalling.

Prior colonisation protects against re-colonisation through Th17-mediated rapid neutrophil recruitment [23]. Hence, although we did not measure the bacterial load in the nasopharynx after the second dose, we would anticipate it is cleared more rapidly than the original inoculum. The ability of repeated doses of nasopharyngeal inoculation to induce stronger immune responses has been previously reported and can be protective even with mutant strains [6,28]. Hence once sufficient bacterial exposure has occurred to induce a primary immune response, further exposure with a second inoculation probably acts as an immunological booster even without prolonged duration of dense colonisation. It is thus possible that administering repeated doses of any of the non-protective mutant strains reported in this work may enhance immunity sufficient to cause protection.

The data presented here directly comparing the several non-protective mutant bacterial strains with their protective parent WT strain aid our understanding of why certain live attenuated strains are able to function as effective vaccines. Duration and/or density of colonisation appear important in determining this, but comparison to other data suggests thresholds clearly vary between different bacterial strains and hosts. For live attenuated strains containing other disabling mutations, sustaining colonisation by inclusion of capsule may be a strategy to enhance the immunogenicity of the non-capsular antigens present in the strain and induce protection against invasive disease.
